# Urine Antibiotic Activity in Patients Presenting to Hospitals in Laos: Implications for Worsening Antibiotic Resistance

**DOI:** 10.4269/ajtmh.2011.11-0076

**Published:** 2011-08-01

**Authors:** Manisone Khennavong, Viengmon Davone, Manivanh Vongsouvath, Rattanaphone Phetsouvanh, Joy Silisouk, Olay Rattana, Mayfong Mayxay, Josée Castonguay-Vanier, Catrin E. Moore, Michel Strobel, Paul N. Newton

**Affiliations:** Wellcome Trust-Mahosot Hospital-Oxford Tropical Medicine Research Collaboration, Mahosot Hospital, Vientiane, Lao PDR; Francophone Institute for Tropical Medicine, Vientiane, Lao PDR; Centre for Tropical Medicine, Nuffield Department of Clinical Medicine, University of Oxford, Churchill Hospital, Oxford, United Kingdom; Faculty of Postgraduate Studies, University of Health Sciences, Vientiane, Lao PDR

## Abstract

Widespread use of antibiotics may be important in the spread of antimicrobial resistance. We estimated the proportion of Lao in- and outpatients who had taken antibiotics before medical consultation by detecting antibiotic activity in their urine added to lawns of *Bacillus stearothermophilus*, *Escherichia coli*, and *Streptococcus pyogenes*. In the retrospective (*N* = 2,058) and prospective studies (*N* = 1,153), 49.7% (95% confidence interval [CI] = 47.4–52.0) and 36.2% (95% CI = 33.4–38.9), respectively, of Vientiane patients had urinary antibiotic activity detected. The highest frequency of estimated antibiotic pre-treatment was found in patients recruited with suspected central nervous system infections and community-acquired septicemia (both 56.8%). In Vientiane, children had a higher frequency of estimated antibiotic pre-treatment than adults (60.0% versus 46.5%; *P* < 0.001). Antibiotic use based on patients histories was significantly less frequent than when estimated from urinary antibiotic activity (*P* < 0.0001).

## Introduction

Antibiotic resistance remains a major public health problem, affecting treatment decisions, patient outcome, health care expenditure, and public perceptions of health care.^1^–^3^ Widespread unregulated provision of antibiotics, dispensing of insufficient doses, reduced adherence to complete dose regimens, and the poor quality of the drug supply are thought to contribute to the spread of antibiotic resistance.^1^–^9^

The Lao PDR (Laos) is situated mostly to the east of the Mekong River and the majority (83%) of the population of 5.2 million people are rural rice farmers with a per capita income equivalent to 326 United States dollars/year.^10^ In comparison to wealthier countries in Asia, there is little information on the clinical epidemiology of infectious disease, although available information suggests that antibiotic resistance levels are relatively low compared with surrounding countries.^10^–^15^ Antibiotics are widely available without prescription at private pharmacies in Laos.^16^–^19^ Dispensing of a single dose or an incomplete course is widespread. The proportion of patients with suspected community-acquired septicemia and meningitis with positive blood or cerebrospinal fluid (CSF) cultures is relatively low and prior antibiotic use may be a contributory factor.^10^,^20^,^21^ To estimate the proportion of patients taking antibiotics before hospital consultation, simple techniques for determining antibiotic activity in patients' urine on “lawns” of reference strains of bacteria have been developed.^22^–^31^ We estimated the proportion of Lao patients who had taken antibiotics before medical consultation by an adaptation of the techniques of Liu and others.^30^,^31^

## Patients and Methods

### Study site and patients.

A retrospective study was conducted using urine collected from patients presenting at Mahosot, Setthathirat, and Phalanxay Hospitals. Mahosot and Setthathirat Hospitals are 365- and 175-bed primary-tertiary care hospitals, in Vientiane, with ∼1,200 and 1,040 admissions/month, respectively.^10^ They are visited by ∼16,000 and 4,500 outpatients/month, respectively. Phalanxay District Hospital is a 10-bed clinic in Savannakhet Province, 105 km northeast of Savannakhet city, southern Laos. Urine was collected before the administration of antibiotics in the hospital and stored as part of the clinical studies at Mahosot and Setthathirat Hospitals investigating the causes of central nervous system (CNS) infections, community-acquired septicemia^10^ and those with suspected typhus, and at Phalanxay District Hospital investigating the non-malarial causes of fever (see Table 1 base; Mayxay M, unpublished data). Those unable to provide urine before administration of antibiotics in the hospital were excluded from the study. Because patients could be included in up to three studies in Vientiane, depending on their clinical presentation, they were allocated in the following order—suspected CNS infection, suspected typhus, and suspected community-acquired septicemia. Therefore, a patient suspected of having all three conditions would be analyzed as a suspected CNS infection. All patients with suspected CNS infection had blood cultures taken and 1.2% of patients were included in all three studies. Patients admitted with suspected typhus had whole blood taken for rickettsial serology and blood culture, those with suspected community-acquired septicemia had blood cultures taken and whole blood for serology and those with clinical evidence for CNS system infections, CSF examination, culture and polymerase chain reaction (PCR), blood cultures, and whole blood for serology. Patients were further investigated and treated according to local hospital practice. Urine samples were collected in sterile plain screw capped 5 mL tubes and stored at −20°C until analysis.

A prospective study was performed between March and July 2005 in Vientiane. All patients attending outpatients on Mondays and Wednesdays during 4 weeks at Mahosot and 8 weeks at Setthathirat Hospitals and all patients admitted to the Adult and Pediatric Infectious Disease Wards (Mahosot Hospital) during 2 weeks and Internal Medicine and Pediatric Wards (Setthathirat Hospital) during 2 weeks were asked to participate. There were no specialized outpatients—all offered a general medical service. Urine samples were collected as above, before the administration of any antibiotics in hospital, and either assayed on the same day, kept in a refrigerator at +4°C if assayed the next day, or in −80°C freezer if assayed within the following 7 days. Visibly fecal-contaminated urine samples were discarded. Outpatients did not undergo specific etiological investigations. Patients were asked whether they had taken any antibiotics in the previous 48 h and, if they had, the name of the antibiotic.

In addition, 20 consenting volunteers who were well and not taking antibiotics provided a “negative control” urine specimen. Ethical clearance was granted by the Ethical Review Committee of the former Faculty of Medical Sciences, National University of Laos, Vientiane, Lao PDR and the Oxford Tropical Medicine Ethics Research Committee. Recruitment to the retrospective studies was through informed oral consent and for the prospective study through informed written consent. Children were defined as < 15 years of age.

### Laboratory procedures.

Sterile filter paper disks were placed on lawns of three reference organisms, an aliquot of urine added to the disks, and after 24 h incubation any zones of inhibition were measured. The reference organisms used were *Bacillus stearothermophilus* (ATCC 7953), *Escherichia coli* (ATCC 25922), and *Streptococcus pyogenes* (ATCC 19615) (not ATCC 19165).^30^ Using a stationary single hole punch 6 mm diameter disks of blank filter paper (Whatman no. 2, Whatman plc, Maidstone, UK) were cut and autoclaved before use. Laminated templates (white circles of paper the size of the Petri dish marked with the numbers 1, 2, 3, 4, and 5 at the outer edge in black) were made and placed below Petri dishes and position 1 marked on the base of the Petri dish. Petri dish (15 cm diameter) agar was of consistent depth (4 mm). Mueller-Hinton agar was used for *E. coli* and *B. stearothermophilus* and Mueller-Hinton agar with 5% goat blood for *S. pyogenes.* The 6 mm disks were placed, using forceps, at positions 1, 2, 3, 4, and 5 (clockwise from position 1) on each dish and gently pressed onto the agar with forceps. Three microliter (µL) aliquots of urine (this volume was determined by experimenting with incremental volumes to give the maximum volume that did not overflow from the paper disk) from five patients were added to individual paper disks on lawns of each organism on two duplicate Petri dishes. Petri dishes were incubated at 35°C for *S. pyogenes* and *E. coli* and 56°C for *B. stearothermophilus* for a timed 24 h, and the zone diameter was read as zero if the growth came up to the disk. If one or both duplicate disks for each reference organism had a visible zone of bacterial inhibition the patient was recorded as having urinary antibiotic activity against that species. The presence of any zone of inhibition around any of the six urine disks/patient defined urinary antibiotic activity.

Quality control of media and organisms was performed weekly on three plates of each media and organism. Instead of applying blank filter paper disks, antibiotic susceptibility testing disks (Oxoid, Basingstoke, UK) were added—*B. stearothermophilus*: penicillin (10 units), chloramphenicol (30 µg), ciprofloxacin (5 µg), erythromycin (15 µg), gentamicin (10 µg); *E. coli:* ampicillin (10 µg), amoxicillin-clavulanic acid (20/10 µg), ceftriaxone (30 µg), chloramphenicol (30 µg), nalidixic acid (30 µg); and *S. pyogenes*: penicillin (10 units), chloramphenicol (30 µg), erythromycin (15 µg), ceftriaxone (30 µg), and vancomycin (30 µg). Minimum inhibitory concentrations (MIC) of the most commonly used antibiotics recorded in the prospective study (below) for the three test organisms were determined after the conclusion of the study by the Etest method.^32^

Our method differed from that of Liu and others^30^,^31^ in that we used 6 mm rather than 8 mm filter paper disks, as hole punches are usually 6 mm, goat rather than sheep blood, and we added a standard volume of urine/disk with a pipette, rather than soaking the disks in urine. Serum samples were stored at −80°C until analysis. Community-acquired septicemia was diagnosed by blood culture^10^ and serum immunofluorescence assays for anti-*Orientia tsutsugamushi* and anti-*Rickettsia typhi* immunoglobulin M (IgM) serum antibodies and microscopic agglutination tests (MAT) for leptospirosis performed.^33^,^34^

### Statistical analysis.

Analysis was performed using STATA version 10 (Stata Corporation, College Station, TX). Categorical variables were compared with χ^2^, Fisher's exact, and McNemar tests and continuous variables by Student's *t*, Mann-Whitney *U*, and Kruskal-Wallis tests as appropriate.

## Results

Urine samples were tested from a total of 3,211 patients, 2,058 (1,844 in Vientiane and 214 in Phalanxay) in the retrospective study and 226 inpatients and 927 outpatients (total 1,153) in the prospective study (Tables 1–3). Quality control of the three test organisms did not show any trend in change in disk diffusion susceptibility through time. The MICs of the most commonly used antibiotics from the prospective study against the test organisms are given in Table 4; none of the 20 healthy volunteers had antibiotic activity detected in their urine.

### Retrospective study.

Admission urine samples from 1,844 patients were collected, at the same time as blood samples for etiological tests and before antibiotic administration to hospital, in Vientiane and from 214 of 229 (93.4%) patients with unexplained non-malarial fever (May–September 2003 and 2004) at Phalanxay. Of the 414 Vientiane patients with suspected CNS infections, 53.9% also had suspected typhus and of 760 with suspected typhus, 96% had suspected community-acquired septicemia. The percentage of patients included, of all those recruited, were 40% of those with suspected typhus and 62% of those with suspected CNS infection. All but 27 of 1,844 (1.5%) Vientiane patients had blood cultures taken and these represented 22% of all those with suspected community-acquired septicemia.

Of the 1,844 Vientiane inpatients, 916 (49.7%, 95% confidence interval [CI] 47.4–52.0) had a zone of inhibition on one or more of the six plates/patient (duplicates of three organism lawns). For the three test organisms, zones of inhibition were detected in one or both plates in 43.8% with *B. stearothermophilus*, 33.3% *S. pyogenes*, and 24.1% *E. coli* (*P* < 0.0001 for all comparisons) (Table 1). There was disagreement between duplicate plates in 3.3% of *B. stearothermophilus*, 1.1% of *E. coli*, and 2.1% of *S. pyogenes*. If only *B. stearothermophilus* had been used, rather than all three organisms, 12.1% (*N* = 111 of 916) of patients with antibiotic activity in their urine would have been falsely classified as negative. The equivalent percentages for *S. pyogenes* and *E. coli* were 33.0% and 51.5%, respectively.

The median (range) age of patients in Vientiane (26 [2 days–93 years] years) was older than in Phalanxay (15 [3–80] years) (*P* < 0.001). Of the three studies in Vientiane median ages differed significantly (*P* = 0.0001) with few children (6%) among those with suspected typhus. The frequency of urinary antibiotic activity was significantly higher in patients in Vientiane (49.7%) than in Phalanxay (37.9%) (*P* = 0.001). In Vientiane, the highest frequency of urinary antibiotic activity was found in patients recruited with suspected CNS infection and suspected community-acquired septicemia (both 56.8%), in comparison to those with suspected typhus (without suspected CNS infection) (37.1%) (*P* < 0.0001). In Vientiane children had a higher frequency of urinary antibiotic activity than adults (60.0% versus 46.5%; *P* < 0.001) but not in Phalanxay (35.5 versus 40.2%; *P* = 0.5).

Clinically significant organisms were cultured from 109 of 1,844 (5.9%) patients who had blood cultures taken (Table 1) and 47 (43.1%) had antibiotic activity in their urine, whereas of 1,735 who were blood culture negative, 869 (50.1%) had antibiotic activity in their urine (*P* = 0.16). Clinically significant bacterial/fungal pathogens were detected by culture or PCR from 43 of 414 (10.4%) with suspected CNS infection. Of the 10 patients with culture-confirmed non-mycobacterial bacterial meningitis 3 (30%) had antibiotic detected in their urine samples, as opposed to 232 of 404 (57.4%) who were culture negative for bacterial pathogens (*P* = 0.08). Of the 10 patients who were PCR positive for CSF bacteria and CSF culture negative, 5 (50%) had antibiotic detected in their urine.

Of the 663 patients with suspected typhus (but not suspected CNS infection), 578 had IFA IgM serum assays, suggesting that 34.8% had scrub typhus and 32.9% had murine typhus. Urinary antibiotic activity was found in 34% of those with community-acquired septicemia and 38% of those with serological evidence for typhus. Of 214 patients recruited at Phalanxay, 25 of 200 (12.5%) had leptospirosis, 13 of 213 (6.1%) had scrub typhus, and none had murine typhus. Of patients with leptospirosis and/or scrub typhus, 9 of 36 (25%) had antibiotic activity in their urine, whereas 72 of 178 (40.1%) of those without evidence for leptospirosis or scrub typhus had urinary antibiotic activity (*P* = 0.08).

### Prospective study.

In the prospective study at two hospitals, 927 of 1,054 (88%) outpatients who attended on the 12 days consented to be interviewed and were able to provide a urine sample (Tables 2 and 3). Nineteen patients (1.8%) did not consent, 28 (2.7%) left before they could give a urine sample and 125 (11.9%) did not complete the questionnaire. Of inpatients 226 of 228 (99%) patients who were admitted during the 2 weeks consented to be interviewed and were able to provide a urine sample (Table 2; 2 [1%] did not give a urine sample before antibiotic administration). Patients attending Mahosot and Setthathirat Hospitals were of similar demography, except that the median age (range) of patients at Mahosot (29 [0.3–87] years) was significantly lower than those at Setthathirat Hospital (33 [0.1–90] years) (*P* = 0.002) (Table 2). A significantly higher proportion of inpatients (40.3%) than outpatients (14.7%) presented with fever (*P* < 0.001).

Of 1,130 patients, 271 (24.0%) stated that they had taken antibiotics in the previous 48 h (of 217, 196 oral, 19 intramuscular, and 2 syrups, Table 2). A higher proportion of patients stated that they took antibiotic in the 48 h before consultation at Mahosot (29.3%) than at Setthathirat (19.8%) (*P* < 0.001), and a higher proportion of inpatients stated that they had taken antibiotics (41.3%) than outpatients (20.0%) (*P* < 0.001). A higher proportion of children were stated (by themselves or parents) to have taken antibiotics (43.5%) than adults (22.4%) (*P* < 0.001). A higher proportion of those who consulted for fever had taken antibiotics (42.2%) than those without fever (19.7%) (*P* < 0.001). There was no significant relationship between smoking (9.2% patients stated that they were current smokers) and the presence of urinary antibiotic activity (*P* = 0.4).

A zone of inhibition around ≥ 1 of the six disks was present from the urine of 36.2% (95% CI = 33.4–38.9) patients (Table 3). There were 1 (0.0009%), 15 (0.01%), and 18 (0.02%) discordant results for the pairs of *E. coli*, *B. stearothermophilus*, and *S. pyogenes* plates, respectively. The proportion of *B. stearothermophilus* plate pairs with evidence for antibiotic activity was (24.8%) and exceeded that for plate pairs of *S. pyogenes* (22.9%) and *E. coli* (11.0%). The proportion of antibiotic activity detected by the three organisms differed significantly (*P* < 0.001). If only *B. stearothermophilus* had been used, rather than all three organisms, 131 of 417 (31.4%) of patients with antibiotic activity in their urine would have been falsely classified as negative. The equivalent percentages for *S. pyogenes* and *E. coli* were 36.7% and 69.5%, respectively.

The proportion of patients who had antimicrobial activity in their urine at Mahosot (33.9%) did not differ significantly from those at Setthathirat Hospital (38.0%) (*P* = 0.2) or between males and females (*P* = 0.1) but the proportion was higher in inpatients (64.6%) than outpatients (29.2%) (*P* < 0.001), higher in children (68.2%) than adults (33.5%) (*P* < 0.001), and higher in those presenting with fever (51.3%) than those without (32.0%) (*P* < 0.001). Antibiotic use based on patient histories was significantly less frequent than when estimated from urinary antibiotic activity (Table 5; McNemar test, *P* < 0.0001).

## Discussion

This investigation suggests that a substantial proportion of patients seeking health care for febrile illnesses in Vientiane and rural southern Laos had taken antibiotics before consultation—in the retrospective analysis in Vientiane and Phalanxay, 49.7% and 37.9%, respectively, and in the prospective Vientiane study 36.2%. Urinary antimicrobial activity has been detected, using similar techniques in 15–67% of diverse patients in diverse locations.^22^,^28^,^29^,^31^,^35^–^38^ In Asia, using similar methodology to that used here, 55.2–67% of Taiwanese patients had urinary antimicrobial activity detected.^22^,^31^ Using *Bacillus subtilis* instead of *B. stearothermophilus,* 38% of 806 febrile Nepalese patients had urinary antimicrobial activity, with no significant relationship between the frequency of blood culture positivity and antimicrobial urinary activity.^39^ Recently, paper disks were impregnated with sera from patients recruited to a study of community-acquired pneumonia/bacteremia in NE Thailand and tested against lawns of *Staphylococcus aureus*–24% of patients had evidence of anti-*S. aureus* activity in their sera. Blood culture yields were significantly lower among patients with evidence for pre-culture antibiotic use.^21^

The discordance between antibiotic activity in urine and patients recall of antibiotic use reflects their uncertainty as to what medicines they have taken, especially when prescriptions are not used, loose pills or capsules are dispensed in small unlabelled plastic bags, and packaging instructions are not in Lao language.

Different patterns of inhibitory zones may help differentiate the antibiotics patients had taken.^30^,^31^ In the Lao retrospective study and in the majority of categories in the prospective study, the urine from more patients inhibited lawns of *B. stearothermophilus* than inhibited the other two bacterial species. This is consistent with the MICs of the three organisms, with *B. stearothermophilus*, having the lowest/joint lowest MICs against the antibiotic panel tested, except for ciprofloxacin and ofloxacin (Table 4). In the retrospective study, inhibition of *E. coli* was most pronounced in urine from patients with suspected CNS infections and less pronounced in children. This could be caused by more fluoroquinolone use in patients with suspected meningitis and less fluoroquinolone use in children, consistent with persisting, but probably unwarranted, concerns of this class of antibiotics in the treatment of serious pediatric infections.

Limitations of the study include that there are no data for the etiological diagnosis for many patients in both retrospective and prospective studies, and we will have underestimated the consumption of rapidly eliminated drugs. We were unable to collect urine from all patients before antibiotic administration and we were unable to determine whether pre-hospital antibiotics were taken in accordance with medical advice. Phalanxay, although in rural Laos, is on a major road to Vietnam and will not be representative of the majority of rural Lao people who are much more isolated.^40^ We used the conservative definition of the presence of any zone of inhibition around any of the six urine disks/patient as indicating urinary antibiotic activity. However, the technique is crude in that the urinary antibiotic activity will depend on the interval between ingestion of medicine and the urine sample, the dose and the time-dependent excretion of antibiotic, and active metabolites in urine. How incubation at 56°C may affect antibiotic-*B. stearothermophilus* interactions seem unknown. For some antibiotics, such as chloramphenicol, little is excreted in the urine.^30^ Therefore, in some settings serum, rather than urine, may be more appropriate sample.^21^

In Taiwanese hospitalized patients receiving multiple drugs, the sensitivity and specificity for detection of antimicrobial activity were for *B. stearothermophilus* 100% and 85.9%; for *S. pyogenes* 94.9% and 94.9%, and for *E. coli* 71.8% and 98.7%, respectively.^30^ There is remarkably little information as to whether septic patients excrete compounds with antimicrobial activity, apart from antibiotics, in urine. Such compounds may cause “false positive” urinary antibiotic activity in hospitalized patients not known to have received antibiotics. Small studies suggest that the urine of healthy volunteers without a recent history of antibiotic ingestion do not contain antibiotic activity,^22^,^30^ but the key information would be whether sick patients with an acute inflammatory response excrete urine with endogenous antibiotic activity. The antibiotic activity of urine from patients fed ketogenic diets was described in 1931^41^ and oral cranberry juice inhibits urinary bacterial growth as does low urinary pH, high urea, ammonia and osmolality, prostatic fluid and semen.^42^–^44^ The bladder mucosa has anti-bacterial activity^45^,^46^ and mediators of acute inflammatory reaction excreted in urine have antibiotic activity.^42^ Ingestion of alcohol, traditional medicines, food substances,^25^ antibiotics from administration to food animals, food additive, fungal growth, and illicit formaldehyde contamination of food could also have antibacterial effects but there is little relevant evidence.^46^–^50^ Lao people suffer from chronic infestation with a diversity of intestinal/biliary parasites^51^ and the recent observation that such infections are associated with heightened oxidative stress in urine^52^ suggests that there may be some associated antibacterial activity. Antibiotic use patterns vary widely between communities and the organisms used in the urinary antimicrobial activity assay will need to be optimized using information about local antibiotic drug use. Medicines may not be labeled or may not contain what they are labeled as containing. Counterfeit medicines containing unexpected anti-infectives are probably widespread and patients may have unexpected antibiotic activity in their urine despite not having knowingly taken antibiotics.^53^ How these diverse factors may have influenced the results of this and previous studies is unknown.

The selection of drug-resistant pathogens depends on a wide variety of factors—the biomass of infecting pathogens, drug dose, host immunity, the relationship between the pharmacokinetic profile of the drug and pharmacodynamic affects on the pathogen, the antimicrobial susceptibility of the pathogen, and the fitness of resistant mutants. The use of antibiotics in the month before an infection was a risk factor for development of systemic penicillin-resistant *Streptococcus pneumoniae* infections in the community.^54^ In Vietnam, a significantly higher frequency of nasal *Haemophilus influenzae*, *S. pneumonia*, and *Moraxella catarrhalis* resistant to ampicillin and/or penicillin was found in children with prior ampicillin and/or penicillin use.^55^ There is evidence from Europe that antimicrobial resistance is related to high antibiotic use^7^,^56^ but such data are sparse from the developing world.^1^,^2^ It remains unclear whether it is excessive drug use per se that is important or inappropriate consumption. Although Laos appears to have lower levels of antibiotic resistance in comparison to adjacent countries,^10^ extended-spectrum β-lactamase-positive *E. coli* and *Klebsiella pneumoniae* clinical isolates have arrived in Vientiane.^14^ It is likely that as the economy improves a greater volume and diversity of antibiotics will be consumed. The high frequency of antibiotic use in the community, as revealed by urinary antibiotic activity, may engender worsening drug resistance.

## Figures and Tables

**Table 1:** Demographic and clinical characteristics of 2,058 patients and the percentage with antibiotic activity detected in their urine in the retrospective examination of inpatients in Vientiane and those with unexplained fever at Phalanxay

Study	% Male	Age/years*	% < 15 years old	No. of urine specimens	No. (%) (95% confidence interval [CI]) with any antimicrobial activity in urine	No. (%) (95%CI) urine samples containing antimicrobial activity according to species of target organism		
						*E. coli*	*B. stearothermophilus*	*S. pyogenes*
All in Vientiane	59.1	26 (0.005–93)^1842^	23.8^1842^	1,844	916 (49.7) (47.4–52.0)	443/1840 (24.1) (22.1–26.0)	805/1839 (43.8) (41.5–46.0)	613/1840 (33.3) (31.2–35.5)
Central nervous system (CNS) infections†	64.5	27 (0.01–84)	23.4	414	235 (56.8) (52.0–61.5)	154/410 (37.6) (32.9–42.3)	212/409 (51.8) (47.0–56.7)	189/410 (46.1) (41.3–50.9)
Typhus suspected without suspected CNS infection‡	60.8	29 (0.33–85)	6.0	663	246 (37.1) (33.4–40.8)	106 (16.0) (13.2–18.8)	202 (30.5) (27.0–34.0)	145 (21.9) (18.7–25.0)
Community-acquired septicemia alone suspected§	54.9	22 (0.005–93)^761^	39.3^761^	763	433 (56.8) (53.3–60.3)	181 (23.7) (20.7–26.7)	389 (51.0) (47.4–54.5)	277 (36.3) (32.9–39.7)
All who had blood cultures taken¶	58.7	26 (0.005–93)^1812^	24.1^1812^	1814	905 (49.9) (47.6–52.2)	433/1810 (23.9) (22.0–25.9)	796/1809 (44.0) (41.7–46.3)	605/1810 (33.4) (31.3–35.6)
All adults	59.1	–	–	1,404	653 (46.5) (43.9–49.1)	317/1401 (22.6) (20.4–24.8)	562/1399 (40.2) (37.6–42.7)	419/1400 (29.9) (27.5–32.3)
All children	58.9	–	–	438	263 (60.0) (55.5–64.6)	126/437 (28.8) (24.6–33.1)	243/438 (55.5) (50.8–60.1)	194 (44.3) (39.6–48.9)
Phalanxay‖	57.5	15 (3–80)	50	214	81 (37.9) (31.4–44.4)	29 (13.6) (9.0–18.1)	67 (31.3) (25.1–37.5)	44 (20.7) (15.2–26.1)^213^
All adults	57.0	–	–	107	43 (40.2) (30.9–49.5)	16 (15.0) (8.2–27.7)	36 (33.6) (24.7–42.6)	22 (20.8) (13.0–28.5)^106^
All children	57.9	–	–	107	38 (35.5) (26.4–44.6)	13 (12.2) (6.0–18.3)	31 (29.0) (20.4–37.6)	22 (20.6) (12.9–28.2)

Denominators are only shown if there are missing values. Number (%) or * Median (range).

† Any patient with clinically suspected community-acquired infection of the CNS without contraindications to lumbar puncture in Vientiane. Clinically significant organisms were cultured from 33 of 414 (8.0%) patients who had cerebrospinal fluid (CSF) culture (*S. pneumoniae* [*N* = 8], *S. suis* [*N* = 1], *K. pneumoniae* [*N* = 1], *C. neoformans* [*N* = 20], and *Mycobacterium tuberculosis* [*N* = 3]). Polymerase chain reaction (20 and Mayxay M, unpublished) assays for *S. pneumoniae*, *Neisseria meningitidis*, and *Haemophilus influenzae* type b in CSF were positive in an additional six patients with *S. pneumoniae* and two each with *N. meningitidis* and *H. influenzae,* giving a total of 43 of 414 (10.4%) of patients having proven bacterial/fungal meningitis.

‡ Any patient with clinically suspected typhus, but without suspected CNS infection, in Vientiane. Thirty-eight (5.7%) had positive blood cultures (growing *S. typhi* [*N* = 19], *E. coli* [*N* = 9], *B. pseudomallei* [*N* = 4], *K. pneumoniae* [*N* = 2], *Salmonella* spp. [*N* = 1], *S. pyogenes* [*N* = 1], *S. aureus* [*N* = 1], and *Aeromonas hydrophilia* [*N* = 1]).

§ Any patient with clinically suspected community-acquired septicemia in Vientiane.

¶ Clinically significant organisms were cultured from 109 of 1,844 (5.9%) patients who had blood cultures taken—*Salmonella enterica* serovar typhi (*N* = 34), *E. coli* (*N* = 23), *Burkholderia pseudomallei* (*N* = 9), *S. aureus* (*N* = 8), *Klebsiella pneumoniae* (*N* = 6), *Salmonella* spp. (*N* = 6), *S. pyogenes* (*N* = 3), *S. pneumoniae* (*N* = 9), *A. hydrophila* (*N* = 1), *Edwardsiella tarda* (*N* = 1), Group B *Streptococcus* (*N* = 1), *S. suis* (*N* = 1), *Neisseria meningitidis* (*N* = 1), *Cryptococcus neoformans* (*N* = 4), and *Penicillium marneffei* (*N* = 2).

‖ Any patient, presenting at Phalanxay District Hospital, who the admitting physician thought may have malaria and performed slide microscopy and/or malaria rapid diagnostic test for *P. falciparum* (Paracheck, Orchid Industries, Goa, India) that were negative.

**Table 2:** Demographic and clinical characteristics of 1,153 patients included in the prospective examination of antibiotic activity in urine at Mahosot Hospital and Setthathirat Hospital and combined

Variable	Mahosot, *N* = 513		Setthathirat, *N* = 640		Both hospitals				
	Inpatients	Outpatients	Inpatients	Outpatients	Inpatients	Outpatients	Adults	Children	Total
No.	90 (18)	423 (83)	136 (21)	504 (79)	226 (20)	927 (80)	1065 (92)	88 (8)	1153
Age/years*	15.5 (0.3–64)	31 (16–87)	32 (0.1–90)	33 (10–84)	24 (0.1–90)	32 (10–87)	33 (16–90)	4 (0.1–15)	31 (0.1–90)
Male gender	51 (57)	159 (38)	60 (44)	226 (45)	111 (49)	385 (42)	449 (42)	47 (53)	496 (43)
Lao Loum ethnicity	74 (82)	399 (94)^422^	121 (89)	479 (95)^503^	195 (86)	878 (95)^925^	999 (94)^1,063^	74 (84)	1073 (93)^1,151^
Civil servant	4 (4)	99 (24)^415^	11 (8)	147 (29)^501^	15 (7)	246 (27)^916^	261 (25)^1,054^	0	261 (23)^1,142^
Farmer	10 (11)	69 (17)^415^	12 (9)	34 (7)^501^	22 (10)	103 (11)^916^	125 (12)^1,054^	0	125 (11)^1,142^
Illiterate	3 (7)^45^	37 (9)	13 (13)^99^	20 (4)^498^	16 (11)^144^	57 (6)^921^	73 (7)	–	73 (7)^1,065^
Primary education	14 (16)	99 (24)^415^	29 (21)	117 (23)^501^	43 (19)	216 (24)^916^	243 (23)^1,054^	16 (18)	259 (23)^1,142^
University/college	7 (8)	38 (9) ^415^	5 (4)	61 (12)^501^	12 (5)	99 (11)^916^	110 (10)^1,054^	0	110 (10)^1,142^
Antibiotics in prior 48 h†	40 (47)^85^	106 (26)^413^	48 (38)^128^	77 (15)	88 (41)^213^	183 (20)^927^	234 (22)^1,045^	37 (44)^85^	271 (24)^1,130^
Presented with history of fever‡	44 (49)	62 (15)^415^	47 (35)	73 (15)^502^	91 (40)	135 (15)^917^	191 (18)^1,055^	35 (40)	226 (20)^1,143^

Number (%) or * Median (range). Denominators are only shown if there are missing values.

† The antibiotics that had been taken were known for 217 (80.1%) patients: ampicillin 135 (62.2%), amoxicillin 26 (12.0%), penicillin 21 (9.7%), doxycycline 9 (4.1%), co-trimoxazole 5 (2.3%), ofloxacin 4 (1.8%), erythromycin 4 (1.8%), tetracycline 2 (0.9%), chloramphenicol 2 (0.9%), and one each (0.5%) for co-amoxiclav, ceftriaxone, cefaclor, ciprofloxacin and kanamycin.

‡ The main discharge diagnoses for outpatients were fever (14.7%), musculoskeletal pain (12.3%), headache (8.6%), cardiovascular disease (7.3%), gastritis (5.8%), and abdominal pain (6.1%). For inpatients the main discharge diagnoses were fever (40.3%), diarrhea (18.1%), and cardiovascular disease (5.3%).

**Table 3:** Frequency of detection of antibiotic activity in urine of 1,153 patients recruited to prospective study

Location	No. of urine specimens	No. (%) (95% confidence interval [CI]) patients who stated that they took antibiotic before	No. (%) (95% CI) with any antimicrobial activity in urine	No. (%) (95% CI) urine samples containing antimicrobial activity according to species of target organism		
				*E. coli*	*B. stearothermophilus*	*S. pyogenes*
Mahosot Hospital						
All patients	513	146^498^ (29.3) (25.3–33.3)	174 (33.9) (29.8–38.0)	61 (11.9) (9.1–14.7)	116 (22.6) (19.0–26.2)	127 (24.8) (21.0–28.5)
Outpatients	423	106^413^ (25.7) (21.5–29.9)	109 (25.8) (21.6–29.9)	53 (12.5) (9.4–15.7)	82 (19.4) (15.6–23.2)	77 (18.2) (14.5–21.9)
Adult infectious disease ward	45	22^42^ (52.4) (37.3–67.5)	36 (80.0) (68.3–91.7)	3 (6.7) (0–14.0)	21 (46.7) (32.1–61.3)	28 (62.2) (48.1–76.4)
Pediatric infectious disease ward	45	18^43^ (41.9) (27.1–56.6)	29 (64.4) (50.5–78.4)	5 (11.1) (1.9–20.3)	13 (28.9) (15.7–42.1)	22 (48.9) (34.3–63.5)
Setthathirat Hospital						
All patients	640	125^632^ (19.8) (16.7–22.9)	243 (38.0) (34.2–41.7)	66 (10.3) (8.0–12.7)	170 (26.6) (23.1–30.0)	137 (21.4) (18.2–24.6)
Outpatients	504	77 (15.3) (12.1–18.4)	162 (32.1) (28.1–36.2)	41 (8.1) (5.7–10.5)	104 (20.6) (17.1–24.2)	94 (18.7) (15.3–22.1)
Adult ward	100	29^92^ (31.5) (22.0–41.0)	54 (54.0) (44.2–63.8)	19 (19.0) (11.3–26.7)	43 (43.0) (33.3–52.7)	27 (27.0) (18.3–35.7)
Pediatric ward	36	19 (52.8) (36.5–69.1)	27 (75.0) (60.9–89.2)	6 (16.7) (4.5–28.9)	23 (63.9) (48.2–79.6)	16 (44.4) (28.2–60.7)
All patients	1153	271^1,130^ (24.0) (21.5–26.5)	417 (36.2) (33.4–38.9)	127 (11.0) (9.2–12.8)	286 (24.8) (22.3–27.3)	264 (22.9) (20.5–25.3)
All adults	1065	234^1,045^ (22.4) (19.9–24.9)	357 (33.5) (30.7–36.4)	115 (10.8) (8.9–12.7)	248 (23.3) (20.8–25.8)	224 (21.0) (18.6–23.5)
All children	88	37^85^ (43.5) (33.0–54.1)	60 (68.2) (58.5–77.9)	12 (13.6) (6.5–20.8)	38 (43.2) (32.8–53.5)	40 (45.5) (35.1–55.9)
All outpatients	927	183^917^ (20.0) (17.4–22.6)	271 (29.2) (26.3–32.2)	94 (10.1) (8.2–12.1)	186 (20.1) (17.5–22.6)	171 (18.4) (16.0–21.0)
All inpatients	226	88^213^ (41.3) (34.7–48.0)	146 (64.6) (58.4–70.8)	33 (14.6) (10.0–19.2)	100 (44.3) (37.8–50.7)	93 (41.2) (34.7–47.6)

Denominators are only shown if there are missing values

**Table 4:** Etest MICs for the three organisms used in the urine assays*

Antibiotic	Minimum Inhibitory Concentration (MIC) ug/mL		
	*E. coli* ATCC 25922	*B. stearothermophilus* ATCC 7953	*S. pyogenes* ATCC 19615
Ampicillin	6	< 0.016	0.023
Amoxicillin	8	< 0.016	0.016
Ceftriaxone	0.094	0.032	0.032
Chloramphenicol	4	6	2
Ciprofloxacin	0.016	0.25	0.25
Co-trimoxazole	0.094	0.004	0.75
Doxycycline	1.5	0.023	0.19
Erythromycin	96	0.38	0.125
Ofloxacin	0.094	0.5	0.75
Penicillin	> 32	0.002	0.032
Trimethoprim	0.5	0.047	> 32

* Minimum inhibitory concentrations (MIC) were determined by the Etest method according to the manufacturers' instructions, with the exception that those for *B. stearothermophilus* were performed at 56°C. Quality control was undertaken using *E. coli* ATCC 25922 as both a test and control organism, along with *Staphylococcus aureus* ATCC 29213 and *Pseudomonas aeruginosa* ATCC 27853 as appropriate, with reference to the manufacturers' instructions and relevant Clinical and Laboratory Standards Institute reference ranges.^32^

**Table 5:** Relationship between antibiotic uses based on patients history and detected urinary antibiotic activity in prospective study. Number (%)

	Urinary antibiotic activity detected	Urinary antibiotic activity not detected	Total
Patient stated that had taken antibiotics in previous 48 h	157 (57.9)	114 (42.1)	271
Patient stated that had not taken antibiotics in previous 48 h	242 (28.2)	617 (71.8)	859
Total	399	731	1,130
